# Wound of the Heart—Recovery

**Published:** 1897-11

**Authors:** 


### WOUND OF THE HEART; RECOVERY.

In these pages we have repeatedly referred to wounds of the heart where patients have lived for some time after their infliction. Their importance from a medico-legal standpoint make every case of interest.

Samuel Prior, in *The Lancet* of October 9, 1897, describes a case in which an iron pin was thrust into the chest in the region of the heart. The exact situation was one and three-fourths inches below and one inch internal to the left nipple. The pin was directed slightly upwards and inwards. It moved vigorously with every beat of the heart and traveled about an inch upwards and downwards every time. On examination the pin was felt to be firmly embedded in the wall of the heart, which was felt tugging at the inner end of the pin. Removal of this peculiar foreign body was not undertaken without some misgivings as to the result. The pin was rotated and gently withdrawn; the part within the chest measured three inches, making a total length of four inches of 3-16-inch iron wire. The patient was collapsed; he had a small, feeble pulse, 64 per minute; respiration was shallow, 24 per minute; and the skin was cold. There was practically no hemorrhage from the wound. No external hemorrhage appeared subsequently to this, and there were no signs of effusion of blood or serum into the pericardium. The patient kept his eyes closed; the pupils were dilated and reacted but slightly to light. When spoken to he would not answer. The wound was dressed antiseptically, and there was no visible suppuration at any time. On the day following the infliction of the wound there was some subcutaneous emphysema round the wound. The physical sign of pneumothorax appeared, but this and the emphysema disappeared again in twenty-four hours. The temperature, pulse and respiration rates rose to the maximum thirty-six hours after the wound was made. The records were as follows: at 7 P. M. the temperature was 100.8 degrees F., the pulse 112, and the respiration 36; at 9 P. M. the temperature was 101 degrees, the pulse 109, and respiration 32; at 11 P. M. the temperature was 101 degrees, the pulse 105, and the respiration 38. The temperature remained slightly febrile for a week and then became normal. His appetite soon returned and he recovered his usual health. Numerous pulse tracings were taken. Soon after the pin was removed the tracing showed a low tension pulse with almost full dicrotism and slight irregularity. On the third day the tracing showed a better tension pulse; the dicrotism was nearly gone. On the fourth day there was still some dicrotism; the tracings showed great amplitude of the percussion wave. Normal tracings were obtained on the succeeding days. There are now (nearly six months after) no signs of pericardial adhesion. The only evidence left of the injury is a reddish linear scar half an inch long, directed downwards and outwards.—*Medicine.*

